# Caffeine improves work durability and physical performance in anaerobic exercises among active adults in Tripoli Lebanon

**DOI:** 10.1186/s13102-021-00334-9

**Published:** 2021-09-02

**Authors:** Zeina Tayba, Yonna Sacre, Randa Attieh, Haider Mannan

**Affiliations:** 1 Holy Spirit University of Kaslik, Jounieh, Lebanon; 2 Western Sydney University, Campbelltown, NSW 2560 Australia

**Keywords:** Active adults, Sport performance, Caffeine consumption, Ergogenic aid, Tripoli

## Abstract

**Background:**

People worldwide have a high intake of caffeine. Active adults are among the group of people who have always been interested in caffeine as an ergogenic aid. This study aims at examining the association between caffeine consumption and perceived performance (aerobic and anaerobic exercises) among active people attending different gyms in Tripoli, Lebanon.

**Methods:**

A cross-sectional study was conducted on 206 participants attending seven gyms in Tripoli. Participants were recruited using simple random sampling for a face-to-face individual interview. Data were collected using a semi-structured questionnaire. Data have been coded, entered, and analyzed using SPSS software.

**Results:**

The prevalence rate of active people practicing both aerobic and anaerobic exercises is 63.6%. More than half of our sample (54.8%) has been working out for a duration of more than 6 months and 84.6% of respondents spend at least 30 min during their workouts. Caffeine consumption was popular in our sample with 92.2% indicating that they use caffeinated products. The findings of this study showed an association between anaerobic exercise and caffeine, perceived as physical performance enhancer and work durability enhancer. Yet no association was found between aerobic exercise and caffeine consumption.

**Conclusions:**

Perhaps, future research could focus on the safe doses of caffeine that could be given for anaerobic exercises to have an ergogenic effect. This could help us to build scientific guidelines for caffeine’s association with sports performance.

## Background

Active adults are among the group of people who always have been interested in ergogenic aids, including caffeine effect on sports performance [1]. Since the 1970s, caffeine has been researched for its role in sports performance [1], yet researches have inconsistent results regarding the effect of caffeine on aerobic and anaerobic exercises. This study will determine the association between caffeine and aerobic/anaerobic exercises, in different gyms in Tripoli- Lebanon. McDaniel et al. [2] revealed in their research, two types of workouts, anaerobic and aerobic exercises which are high and low intense respectively. Khoury and Jonville [3] studied the prevalence of people exercising in different gyms in Beirut city. The results have shown that out of 512 participants, most of them were men (60.9%). Those participants aged between 20 and 50 years exercise routinely 3–5 times per week [3]. People in Lebanon, especially university students, tend to be addicted to caffeine consumption. A study has shown that 97% of 800 students from different universities in Lebanon drink around 193 mg of caffeine per day [3, 4]. Sachse et al. [5] studied the individual variation following caffeine consumption. The results have shown that some individuals can metabolize caffeine faster than others, resulting in better sports performance [6, 7].

Kaczka et al. [8] have demonstrated that supplements including caffeine have a positive effect on anaerobic activities, especially in delaying fatigue and increasing strength. Madden et al. [9] also found that the rating of perceived exertion and physical performance was higher in the group of individuals ingesting caffeine. McDaniel et al.’s [2] theory suggested that caffeine consumption had a direct effect on the central nervous system by increasing alertness and decreasing fatigue. Caffeine consumption had a positive association with aerobic and anaerobic exercises [2, 10]. McDaniel et al. [2] have found that caffeine consumption increases productivity while performing aerobic and anaerobic exercises. Yet, it had no effect on reducing fatigue after soccer game [11], while physical performance is only improved after caffeine consumption when performing anaerobic activities [2, 9]. Since the literature has inconsistent results regarding the effect of caffeine on anaerobic and aerobic exercises, this study is concerned with determining the association between caffeine and aerobic/anaerobic exercises. We hypothesize that caffeine consumption has positive association with both aerobic and anaerobic exercises.

## Methods

### Study design

This Cross-sectional study conducted between caffeine consumption and physical activity aims to investigate the impact of caffeine consumption on perceived performance among a group of adults that are sustaining regular gym activity in Tripoli.

### Sample recruitment and selection

#### Recruitment of gyms

We employed a random sampling technique by visiting all 10 gyms in the city centre of Tripoli, Lebanon, and recruiting adults who were active in these gyms to take part in the study. All participants in these gyms had access to aerobic and anaerobic activities. Our exclusion criteria include gyms with a population fewer than 30 individuals as well as single-sex gyms. Accordingly, two gyms were excluded for not fitting these criteria. Moreover, one gym’s administration refused to participate in the study. As such, a total of 7 gyms meeting these criteria were included. The population size of all individuals who use these gyms is 1750.

#### Recruitment of population

Our population included all adults who are physically active in the area of Tripoli, Lebanon. Full lists of customers were provided by each gym and we randomly picked a sample from each. One gym refused to provide a list of its customers, therefore, we estimated the total number based on the size and importance of the gym in the area, and the population size of similar gyms. The population size of all individuals who use these gyms is 1750. Based on the method introduced by Krejcie and Morgan (1970), and cited in Goyette [12, 13], we calculated a minimum sample size of n = 193 for detecting the effect of caffeine on aerobic/anaerobic exercise, while accounting for 10% non-response this number increased to 214. A total of 31 individuals from each gym were invited to participate in the study.

### Exclusion and inclusion criteria of the sample

All individuals who consume products containing caffeine and perform aerobic and/or anaerobic exercise in different gyms in Tripoli- Lebanon were included in our sample. Participants not meeting these criteria were excluded. All participants voluntarily agreed to take part in the study research and understood what is required of them.

### Data collection

Data was collected inside the targeted gyms by the main student. The student visited gyms for 8 days between 5 and 8 pm, which were indicated as the busiest hours by the gym administrations. In each gym, the student stayed at the reception desk and randomly invited participants who had finished their exercise session to fill in the survey in the English language. Data collection took place after the consent and approval of the key informants, i.e. gym administrations were obtained. On average, participants spent 15 min filling the survey. Some participants completed the survey on their own and others sought the support of the student to understand and answer some questions. There were 206 gym goers who filled the survey, giving a response rate of 94.9%.

### Data collection tool

Our main data collection tool was a questionnaire that surveys participants about their caffeine consumption habits, their physical activity, and their perception of the association between these two variables [14, 15]. We chose questionnaires due to the feasibility and convenience of their administration, as well as their low cost and efficiency. Additionally, questionnaires are a suitable tool for investigating our research questions using a quantitative approach given that they can be conducted in large numbers, which increases the reliability and confidence in our results.

### Data analysis

Survey data were entered into SPSS, version 22, by the main student. Moreover, various types of statistical analysis were done using SPSS, depending on the nature and content of the questions and answers. Frequencies and descriptive statistics were performed for the first two sections of the questionnaire (socio-demographic Questions and Health Questions), duration of aerobic exercise, frequencies of aerobic and anaerobic exercise, and frequency of caffeine consumption.

In addition to descriptive analysis, reliability test, factor analysis, and correlations were conducted to assess the association between caffeine consumption and aerobic and anaerobic exercises. Since our 16 items related to individual beliefs about the effect of caffeine reflect different dimensions underlying caffeine beliefs, we can proceed with conducting a factor analysis. However, for confirmation, the Kaiser–Meyer–Olkin (KMO) test and Bartlett’s test for sphericity were conducted on the 16 items [14]. The KMO test assesses how suitable the data is for factor analysis and it is a measure of the proportion of variance among items that might have common variance. The larger the proportion, the more suitable the data is for factor analysis. KMO values between 0.8 and 1 indicate that the sampling is adequate and factor analysis is meritorious or marvelous [14].

### Independent and dependent variables

There are 3 main dependent variables (DVs) that will be assessed in this study: (1) aerobic exercise frequency, (2) aerobic exercise duration, and (3) anaerobic exercise frequency. These three variables are assessed first, based on two questions that ask respondents to describe how frequently they practice each type of exercise, on a scale from 0 to 7 (0 = rarely/never and 7 = multiple sessions day). Second, the aerobic duration of exercise was assessed asking respondents to describe their aerobic duration with a time range of less than 30 min to more than 90 min (< 30 min, 30–60 min, 61–90 min and > 90 min). The independent variables (IV) that will be assessed in this study are related to caffeine questions that ask respondents to rate their level of agreement on the following items using the Likert scale: “caffeine improves athletic performance”, “workouts are better after having caffeine”, “caffeine increases my motivation to work”, “I would be unable to function without caffeine”, “I can exercise longer if I have caffeine”, “caffeine picks me up when I’m feeling tired”, “caffeine improves my mood”, “caffeine improves my concentration”, “caffeine helps me work over a long period of time”, “caffeine improves my attention”, “caffeine makes me feel more energetic”, “I feel less sleepy after having caffeine”, “caffeine at any time throws off my sleep”, and “caffeine makes me feel more alert”.

## Results

### Demographics

Results showed that out of the total of 214 invited participants, 206 participants covering 7 gyms participated in our study. Our study yielded a response rate of 96.26% across gyms. Participants (n = 206) included 151 males (73.3%), 54 females (26.2%) and 1 not stating gender. The majority of our sample (76.1%) were between the ages of 18 and 35, 14.1% of it were below 18, and the remaining 9.8% were 36 and above.

### Description of exercise type and frequency among the study sample

Our results suggest that respondents had diverse exercise types, with 63.6% of the sample reporting that they practice both aerobic and anaerobic exercise, 18.4% reporting that they practice only anaerobic exercise, and 14.6% saying that their exercise is aerobic. Across these diverse exercise types, 54.8% of the sample has been working for a duration of more than 6 months, indicating that the sample is generally experienced with athletic performance and physical activity. Additionally, 33.5% which represents the majority of our respondents work out for a duration of 30–60 min, Also, 30.9% of respondents spend 61–90 min during their workouts. Consequently, 14.9% of the participants’ report that they work out for less than 30 min and 19.1% of them practice for more than 90 min indicating that the participants work out for a good amount of time.

For most of the following analyses, participants will be distributed in two pools, those who practice aerobic exercise (n_1_ = 161) and those who do anaerobic exercise (n_2_ = 169). The mean frequency of exercise among pool 1 (aerobic exercise) is 4.31 days/week (SE = 0.334) and that among pool 2 (anaerobic exercise) is 4.40 days/week (SE = 0.143).

**Fig. 1:**
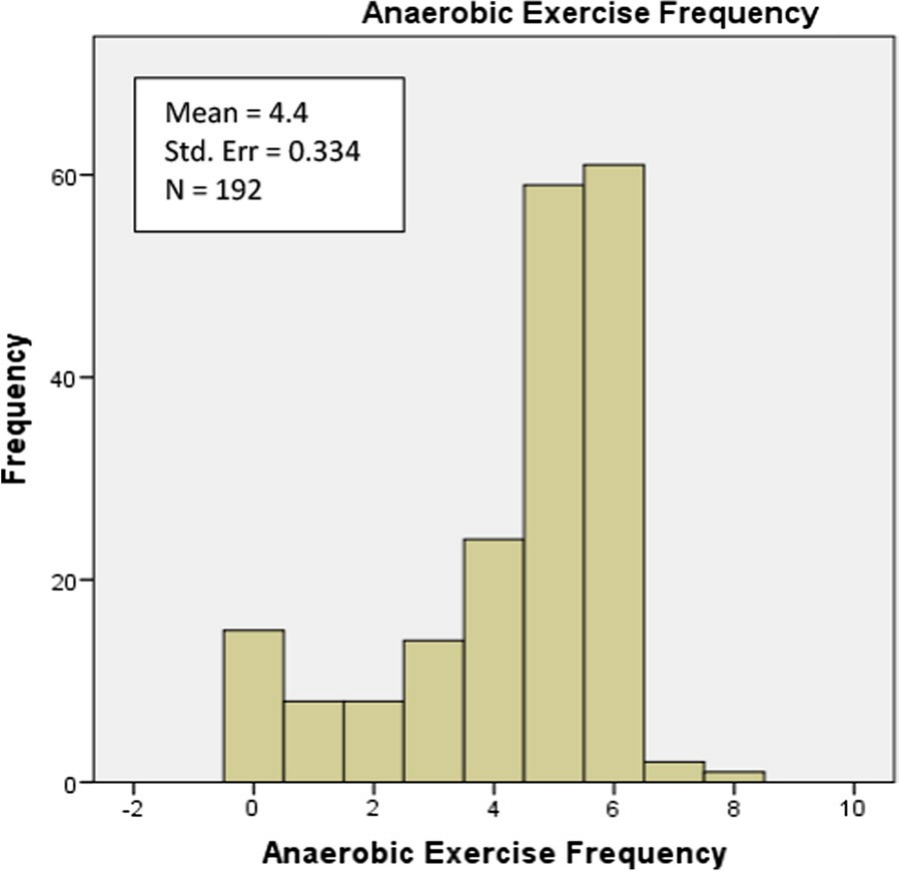
Anaerobic exercise frequency of the study sample

**Fig. 2:**
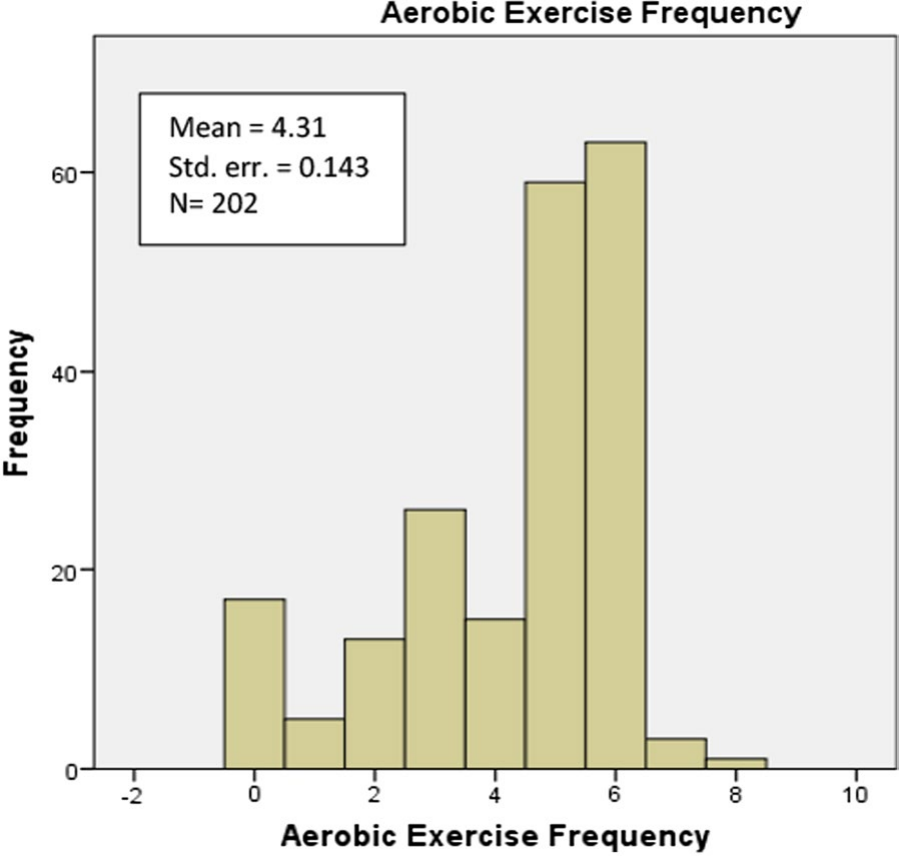
Aerobic exercise frequency of the study sample

The majority of our participants from both pools, anaerobic exercise and aerobic exercise, report that they exercise for 5–6 days/week. As shown in Fig. 1, 30.7–31.8% practice anaerobic exercise 5–6 days respectively. Additionally, 29.2–31.2% practice aerobic exercise 5–6 days respectively (Fig. 2).

### Individual beliefs about the effect of caffeine

Caffeine consumption was popular in our sample, with 92.2% indicating that they use a certain caffeinated product, most commonly coffee, soft drinks, and tea. The caffeine expectancy questionnaire included statements related to the influence of caffeine and asked participants to rate their level of agreement with the statements. Table 1 shows that participants agreed the most with the statement “caffeine picks me up when I am feeling tired” (*Mean* = 3.55, *SE* = 0.252) and disagreed the most with the statement “I am easily stressed after having caffeine” (*Mean* = 2.41, *SE* = 0.172) suggesting that the sample is experienced with similar knowledge about the effect of caffeine.

**Table 1:** Individual beliefs about the effect of caffeine consumption

	N	Mean	SE
1. Caffeine picks me up when I am feeling tired	199	3.55	0.252
2. I feel less sleepy after having caffeine	193	3.35	0.241
3. Caffeine improves my attention	195	3.38	0.242
4. Caffeine makes me feel more energetic	198	3.54	0.252
5. Caffeine makes me feel more alert	197	3.53	0.252
6. Caffeine improves my concentration	194	3.45	0.248
7. I can work longer if I have caffeine	194	3.25	0.233
8. Caffeine improves my athletic performance	194	3.23	0.232
9. Workouts are better after having caffeine	194	3.39	0.243
10. I can exercise longer if I have caffeine	197	3.10	0.221
11. Caffeine increases my motivation to work	194	3.36	0.239
12. Caffeine at any time of day throws off my sleep	194	3.06	0.241
13. I am easily stressed after having caffeine	197	2.41	0.172
14. Caffeine makes me feel nervous	195	2.72	0.195
15. I would be unable to function without caffeine	194	2.53	0.182
16. Caffeine improves my mood	194	3.47	0.249

### Reliability test

The reliability coefficients were conducted for the caffeine questions (independent variables) that consisted of 16 items. The resulting Cronbach’s alpha was equal to 0.901 which is above 0.7 demonstrating a good level of reliability. Scores demonstrated an acceptable level of reliability. Thus, caffeine questions are consistent, all reliably measure the same independent variable related to individual beliefs of caffeine consumption.

### Factor analysis

We found that KMO is 0.887 implying that the sampling is adequate and factor analysis is meritorious or marvelous. The Bartlett’s test was significant (*p* < 0.0001) indicating that the items were correlated and therefore suitable for data dimension reduction and hence factor analysis may be useful. Thus, the results of both KMO and Bartlett’s tests agreed that a factor analysis can be conducted.

**Fig. 3:**
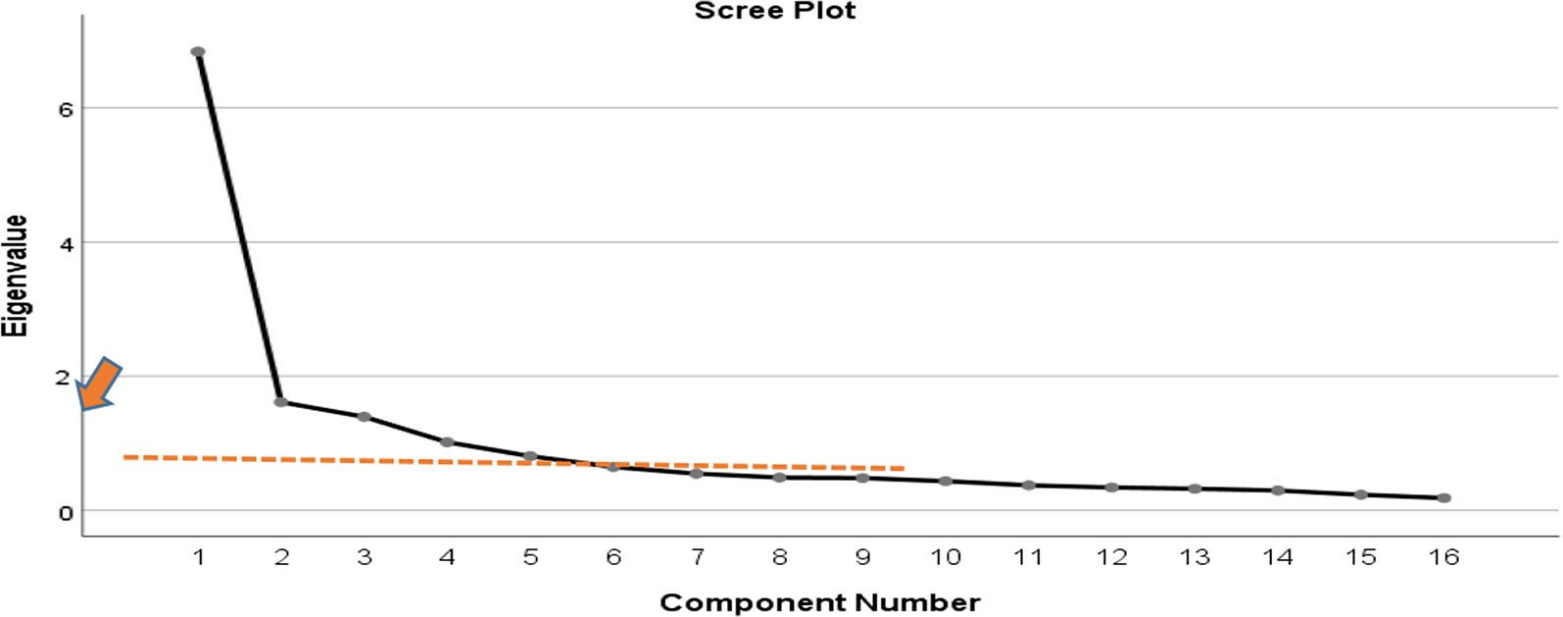
Factor analysis results of caffeine questions

The output in (Fig. 3) mainly shows the presence of the first, second and third factors as influential points with well-presented angles. Therefore, we conclude that the data may be reduced to three factors only which are factor 1 related to physical performance, factor 2 related to work durability, and factor 3 related to productivity.

In the following Table 2*,* we show the results obtained from the factor analysis with the percentage of variances and the eigenvalues obtained for all factors. The first four factors have a total of 67.8% cumulative variance. The first three factors were included, and we excluded the fourth one since it reflects a small number of items. In addition, items underlying this factor were detected as outliers.

**Table 2:** The obtained factors with total variances explained in descending order

Component	Initial eigen values		
	Total	% of Variance	Cumulative %
1. Caffeine picks me up when I’m feeling tired	6.840	42.747	42.747
2. I am easily stressed after having caffeine	1.612	10.077	52.824
3. Caffeine improves my athletic performance	1.394	8.713	61.537
4. I feel less sleepy after having caffeine	1.015	6.343	67.879
5. Caffeine improves my mood	.806	5.038	72.917
6. Workouts are better after having caffeine	.645	4.029	76.946
7. Caffeine increases my motivation to work	.545	3.408	80.354
8. Caffeine at any time throws off my sleep	.488	3.048	83.403
9. Caffeine makes me feel nervous	.480	3.000	86.403
10. Caffeine makes me feel more alert	.433	2.709	89.112
11. Caffeine improves my concentration	.372	2.325	91.437
12. Caffeine helps me work over long period of time	.340	2.122	93.559
13. I would be unable to function without caffeine	.321	2.008	95.566
14. Caffeine improves my attention	.294	1.839	97.406
15. I can exercise longer if I have caffeine	.232	1.452	98.858
16. Caffeine makes me feel more energetic	.183	1.142	100.000

Extraction method: principal component analysis

The eigenvectors for the first three factors, are presented in the following Table 3. All the values of the first vector are positive, hence the vectors were sorted by the second and then by the third vectors. Based on the eigenvectors values, four groups of variables were identified. The first group of items include: 3 (caffeine improves my athletic performance), 6 (workouts are better after having caffeine), 7 (caffeine increases my motivation to work), 13 (I would be unable to function without caffeine) and 15 (I can exercise longer if I have caffeine).

**Table 3:** The eigenvectors of the first three factors ordered

	Factor 1	Factor 2	Factor 3
Item6: Workouts are better after having caffeine	0.710	− 0.258	0.311
Item3: Caffeine improves my athletic performance	0.650	− 0.226	0.403
Item13: I would be unable to function without caffeine	0.456	− 0.225	0.263
Item7: Caffeine increases my motivation to work	0.778	− 0.217	0.172
Item15: I can exercise longer if I have caffeine	0.732	− 0.161	0.260
Item5: Caffeine improves my mood	0.693	− 0.146	− 0.075
Item1: Caffeine picks me up when I’m feeling tired	0.654	− 0.122	− 0.342
Item16: Caffeine makes me feel more energetic	0.766	− 0.050	− 0.021
Item14: Caffeine improves my attention	0.790	− 0.023	− 0.146
Item11: Caffeine improves my concentration	0.786	− 0.012	− 0.257
Item4: I feel less sleepy after having caffeine	0.641	0.249	− 0.336
Item10: Caffeine makes me feel more alert	0.702	0.266	− 0.277
Item8: Caffeine at any time throws off my sleep	0.474	0.489	− 0.356
Item12: Caffeine helps me work over long period of time	0.752	0.049	0.038
Item2: I am easily stressed after having caffeine	0.265	0.582	0.592
Item9: Caffeine makes me feel nervous	0.251	0.787	0.299

Extraction method: principal component analysis

The second group of items with values of vectors 2 and 3 as negative include: 1 (caffeine picks me up when I’m feeling tired), 5 (caffeine improves my mood), 11 (caffeine improves my concentration), 12 (caffeine helps me work over a long period of time), 14 (caffeine improves my attention) and 16 (caffeine makes me feel more energetic). The third group of items include 4 (I feel less sleepy after having caffeine), 8 (caffeine at any time throws off my sleep) and 10 (caffeine makes me feel more alert). The fourth group of items with all values positive include 2 (I am easily stressed after having caffeine) and 9 (caffeine makes me feel nervous).

### Correlation analyses

These were carried out to show the association of the three dependent variables; aerobic exercise frequency, aerobic exercise duration, and anaerobic exercise frequency with the three independent variables based on the caffeine questions; physical performance, work durability, and productivity.

Results in Table 4 revealed the correlation between the variables. Aerobic variables were not correlated with any of the independent variables (all p-values were not significant) suggesting that caffeine has no association with aerobic activities when perceived as a physical performance enhancer, work durability enhancer, or productivity enhancer. However anaerobic exercise frequency has a positive significant correlation with physical performance (*p* = 0.05) and work durability (*p* = 0.032), but doesn’t affect productivity.

**Table 4:** Correlation coefficients and the corresponding p-values between independent and dependent variables

	Aerobic exercise frequency	Aerobic exercise duration	Anaerobic exercise frequency
*Physical performance*			
Correlation coefficient	0.067	− 0.012	.145*
*P* value	0.355	0.873	**0.050**
*Durability*			
Correlation coefficient	0.096	0.018	0.157*
*P* value	0.182	0.806	**0.032**
*Productivity*			
Correlation coefficient	− 0.009	− 0.019	0.049
*P* value	0.904	0.805	0.508

*p* ≤ 0.05 indicating significantly different from zero at 5% type 1 error

## Discussion

This cross-sectional study was conducted to examine the association between caffeine consumption and sports performance (aerobic and anaerobic exercises) among active people attending gyms in Tripoli, Beirut. Thus, we studied the relation between the independent variable (IV), “caffeine consumption” and the dependent variable (DV), “physical activity” (aerobic and anaerobic exercises).

Unlike our hypothesis revealing that caffeine consumption has a positive association with aerobic and anaerobic exercises, our results have shown a significant association between anaerobic exercise and caffeine when perceived as a physical performance enhancer and work durability enhancer, nevertheless, no association has been found between anaerobic exercise and caffeine when perceived as a productivity enhancer. In addition, no association has been shown between aerobic exercise and caffeine when perceived in different aspects: physical performance enhancer, work durability enhancer, or productivity enhancer.

Most of our sample were male (73.3%) active exercisers. This result converges with those which studies the prevalence of people exercising in different gyms of Beirut [3]. Their results have shown that most of the 512 participants were male (60.9%). This results suggest that men in Lebanon are more physically active compared to women. These results are also homogenous with the US population, which revealed that 20.6% of American adults were physically active. Out of them, 23.4% were men and 17.9% were women [15].

The majority of our sample (76.1%) were between the ages of 18 and 35, 14.1% of it were below 18, and the remaining 9.8% were 36 and above. US population is also most likely to be active between the ages of 18 and 24 suggesting that younger people are more likely to be physically active than older people [16].

The finding that caffeine consumption was very high (92.2%) among the gym goers in this study is similar to a study done on different university students in Lebanon which indicated that 97% of 800 students used caffeine products [4]. Despite the literature showing a positive association between caffeine consumption and both aerobic and anaerobic activities [2], our results showed the absence of significant association between aerobic exercises and the three factors for the caffeine consumption variable: (1) caffeine as a physical performance enhancer, (2) caffeine as work durability enhancer and (3) caffeine as a productivity enhancer. Yet, a positive association was shown between anaerobic exercises and factor 1 (caffeine as physical performance enhancer) and factor 2 (caffeine as durability enhancer) but no association with caffeine as a productivity enhancer. These results diverge from Davis and Green [10] who showed in their research an ergogenic effect of caffeine on both anaerobic and aerobic exercises. In addition, caffeine has an ergogenic effect for almost all aerobic activities like running, tennis, walking and swimming, and anaerobic exercises [2]. In addition, our data on caffeine consumption and exercise (aerobic and anaerobic) were collected based on past exposure and thus our findings could be affected by recall bias. This type of bias may underestimate the measure of association and reduce the probability of finding a significant association [9].

On the other hand, randomized experimental studies have found that the intake of a moderate dose of caffeine has an ergogenic effect on male ice hockey players [9]. Moreover, it is important to mention that caffeine acts differently between individuals due to genetic variations. For example, some individuals can metabolize caffeine metabolites faster than others, leading to a better effect on physical performance [7]. Salinero et al. [17] also emphasized on genetic variation between individuals regarding caffeine metabolites, linking it with the type of exercise, habituation of caffeine and the level of training. Our statistical results are based on analyzing subjective answers from participants regarding perception towards caffeine consumption as a physical performance enhancer, work durability enhancer, and productivity enhancer, which can be influenced by reporting bias.

Another divergent finding was experimental studies which reported that caffeine has no effect on reducing fatigue after soccer game [11]. However, our results revealed that more than 50% of our participants, in the caffeine expectancy questionnaire, agreed that caffeine picks them up when they are feeling tired (*Mean* = 3.55, *SE* = 0.252). Thus, our sample which represents active people from Tripoli consumes caffeinated products mostly to reduce their fatigue.

Our findings are convergent with a double-blind, placebo-controlled randomized cross over study on anaerobic activities. Madden et al. [9] revealed that perceived effort is greater in the caffeine group compared to the placebo suggesting that caffeine could increase overall physical performance in anaerobic activities. Moreover, McDaniel et al. [2] showed a 4.2 s enhancement in anaerobic activities in the caffeine group compared to placebo. This is coincident with our findings that anaerobic activities have a positive association with caffeine as an overall physical performance enhancer. Furthermore, our results have shown that caffeine when perceived as a work durability enhancer is found to be positively associated with anaerobic exercise which is coincident with the American Alliance for Health theory that caffeine has a direct effect on the central nervous system to increase alertness and decrease fatigue [2]. Also, a randomized double-blind study revealed that caffeine-containing supplements have a positive effect on anaerobic exercises especially in delaying fatigue and increasing strength [7].

Our findings suggest that caffeine has no association with aerobic and anaerobic exercises when perceived as a productivity enhancer. This differs from the literature which revealed that caffeine increases adrenaline level resulting in better productivity when performing both aerobic and anaerobic activities [2].

This study has some strengths and limitations. First of all, the questionnaire included 16 items on the attitudes towards caffeine and physical exercise, which by conducting the reliability test was found to be reliable. This consistency in caffeine belief could be due to our homogenous sample where witnessing drastic variations and ethnic differences are low [7]. Additionally, the main added value of this study is targeting a new population, which sheds light on how generalizable previous findings can be to diverse populations. Also, this observational research study could be the foundation of experimental research that further investigates the impact of caffeine consumption on physical activity and athleticism at a larger scale. In other words, the findings of this study regarding the association between caffeine and physical performance could guide future research by highlighting the associations that should be tested through experimental manipulation. Such studies can lead to more rigid conclusions on the effect of caffeine on physicality. Nevertheless, this research suffers from some limitations, mainly due to not assessing dose-exposure relationships between caffeine uptake and aerobic and anaerobic exercises, its observational nature in terms of study design and reliance on self-report. Some questions require participants to remember their physical activity and caffeine consumption routines, therefore are prone to recall bias. In addition, social desirability bias could alter the answers to questions related to physical performance, health, and diet. Furthermore, it would have been better to categorize the levels of physical activity according to the Physical Activity Level (PAL) scale. Also, the sample may not be representative of the target population as the recruitment was limited to only 7 gyms where our target population of active Lebanese participants in Tripoli were mostly found. Finally, the respondents’ answers to the caffeine beliefs scale could be shaped by common caffeine consumption stereotypes and misconceptions, rather than their personal experiences resulting from consuming caffeine. As such, factor 1 (physical performance enhancement), factor 2 (durability enhancement), and factor 3 (productivity enhancement) may be indicators of caffeine beliefs that are common among the targeted sample rather than indicators of the effects of caffeine on respondents’ physical activity. Previous studies utilized various data collection tools, ranging from surveys to experimental manipulation, to address the questions on caffeine consumption and physical activity. However, this study is observational, using questionnaires as the main source of data. This study will add value to the literature by surveying the entire Lebanese population and, accordingly, guiding further experimental research.

## Conclusion

Our results did not fully support the hypotheses that caffeine consumption has positive association with both aerobic and anaerobic exercises. While our results have shown that anaerobic exercise has positive association with caffeine when the latter is perceived as a physical performance enhancer, and work durability enhancer, no association is found between anaerobic exercise and caffeine when perceived as a productivity enhancer. In addition, aerobic exercise has no association with caffeine when perceived as a physical performance enhancer, work durability enhancer, or productivity enhancer. Perhaps, future research could focus on the safe doses of caffeine that could be given for anaerobic exercises to have an ergogenic effect. This could help us to build scientific guidelines pertaining to caffeine’s association with sports performance.

## Data Availability

The data that support the findings of this study are available from ZT but restrictions apply to the availability of these data, which were used under license for the current study, and so are not publicly available. Data are however available from the authors upon reasonable request and with permission of ZT.

## Abbreviations

PAL: Physical activity level
US: United States
REC: Research Ethics Committee
USEK: Holy Spirit University of Kaslik
IV: Independent variable
DV: Dependent variable
